# Editorial: An International Journey into the Research Progress of Pediatric Cardiology

**DOI:** 10.3390/children10020321

**Published:** 2023-02-08

**Authors:** Massimo Mapelli, Paola Zagni

**Affiliations:** 1 Centro Cardiologico Monzino, IRCCS, 20138 Milan, Italy; 2 Department of Clinical Sciences and Community Health, Cardiovascular Section, University of Milan, 20122 Milan, Italy; 3 Terapia Intensiva Neonatale, Ospedale Fatebenefratelli P.O. Macedonio Melloni, Via Macedonio Melloni 52, 20129 Milan, Italy

The first time we met was in the pediatric ward of St. Mary’s Lacor Hospital, a 600-plus-bed nonprofit hospital located in Gulu, Northern Uganda, one of the poorest areas in Sub-Saharan Africa. 

At that time, we were just two young residents (in cardiology and pediatrics) who had decided to spend 6 months of their training period caring for Ugandan patients to broaden their personal and professional knowledge. I remember Paola running up to me that day and saying: “Mapi (that was what she called me), I really need to introduce you to this child we are treating for a suspected endocarditis. He has a very funny expression, and he is so nice!”

**Figure 1:**
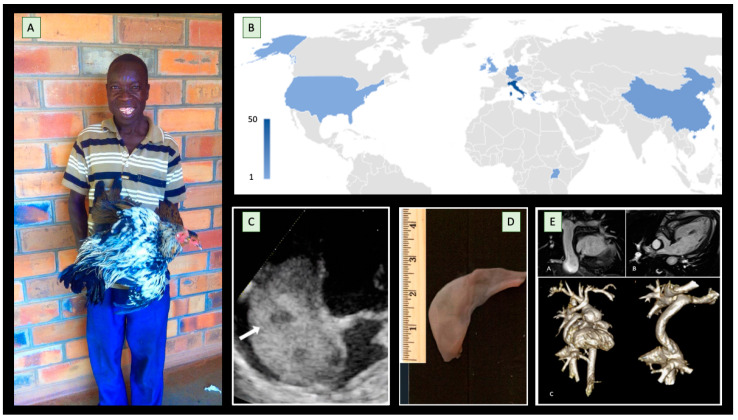
The international journey of pediatric cardiology. (**A**) Gulu, Northern Uganda—after a difficult diagnosis made to his son, the father brings a live chicken as a gift to the doctors (photo obtained with permission) [1]; (**B**) the map represents the geographic origin of the 99 authors of the articles in this Special Issue “Research Progress in Pediatric Cardiology”. Despite the small number of articles [13], several continents are represented in this collection; (**C**) a Wilms’ tumor with intracardiac extension in a young patient undergoing echocardiographic screening [3]; (**D**) the anatomopathological specimen of the excised membrane in a difficult case of cor triatriatum dexter presenting with persistent cyanosis [5]; (**E**) 3D whole-heart cardiac magnetic resonance images allow a comprehensive and detailed evaluation of intracardiac and extracardiac morphology in patients with Fontan circulation [6].

Eventually, it turned out that the child had a rather rare syndrome (Williams Syndrome), characterized by the association of certain congenital cardiological abnormalities (e.g., supravalvular aortic stenosis, pulmonary artery stenosis); a peculiar face, termed “elfin face”; and an excessively sociable behavior (“cocktail behavior”). This diagnosis, particularly brilliant in an environment with limited resources and therefore lacking the main diagnostic tools usually used in such cases, earned us a much-appreciated gift from the patient’s relatives (Figure 1A) and became the clinical case [1] that opens this Special Issue called “Research Progress of Pediatric Cardiology”, a series of 13 articles that provide original contributions from nearly 100 international authors about pediatric cardiology (Figure 1B).

That African experience, in addition to being fruitful from a human and personal point of view, also proved important in terms of clinical research and data collection. In this Special Issue alone, two more papers are derived from those months south of the Equator. The first [2] is an accurate description of an echocardiographic screening campaign for rheumatic heart disease conducted in a Ugandan orphanage, in which methods of execution and results are described in the evaluation of 163 children (between 5 and 14 years of age) who lived in a Ugandan orphanage in the middle of an area endemic for rheumatic disease. The second [3] reports the extraordinarily high prevalence (19%) of the intracardiac extension of Wilms tumor in a pediatric cohort (Figure 1C). Both show how the same cardiac diseases can have very different presenting features depending on the region of the world where they occur. This Special Issue also contains two other clinical cases with a congenital heart disease focus. In the first study by Pecoraro et al. [4], the authors describe a rare case of persistent superior vena cava with occasional oxygen desaturation in a 7-year-old child, highlighting how this heart disease can also occur in school-aged children. The second case, by Picciolli et al. [5], underlines the difficulties in the differential diagnosis of an infant with cyanosis unresponsive to treatment who required urgent cardiac surgery (Figure 1D). The topic of congenital heart disease, one of the prevalent topics in the field of pediatric cardiology, is also covered in two review articles included in this Special Issue. Moscatelli et al.’s manuscript [6] specifically addresses the role of cardiovascular imaging in the follow-up of patients with Fontan circulation (Figure 1E). The Fontan procedure and its modifications have led to a substantial improvement in the survival rates of patients with a variety of types of complex congenital heart disease. However, despite this significant amelioration in the prognosis over the years, Fontan patients are still exposed to several cardiovascular and systemic complications. The authors showed how cardiovascular imaging can play a key role in this context, allowing for the early identification of complications, with important prognostic repercussions. Specifically, even if echocardiography remains the first-line imaging modality for serial evaluation, they described the increasing role of cardiovascular magnetic resonance and cardiac computed tomography. The other review article [7] by Syamasundar Rao deals with double-inlet left ventricle, a condition in which there is a single functioning ventricle (most commonly with a left ventricular structure), and this chamber receives both atrioventricular valves and is connected to an outlet chamber with the morphological features of the right ventricle. In the review, the most important diagnostic and therapeutic options available are carefully described, and the paper concludes that double-inlet left ventricle can be successfully diagnosed with echo-Doppler studies, with this heart anomaly being effectively treated with the currently prevailing medical, catheter interventional, and surgical treatment practices.

In Bassareo et al.’s paper [8], the authors describe the results of a retrospective study involving 107 patients with a perimembranous ventricular septal defect to test the role of oxygen content in the defect’s spontaneous closure, showing how the self-resolution of perimembranous ventricular septal defects seems to rely on numerous factors, including oxygen content, which is likely to promote cell proliferation as well as tissue regeneration.

In addition to papers on congenital heart disease, this Special Issue also includes data presented by Thajer et al. [9], who showed how a screening strategy can be useful to detect familial hypercholesterolemia in a population (n = 512) of pre-school children considered at high risk based on a questionnaire on hypercholesterolemia, cardiovascular events in their family history, or the presence of xanthomas or xanthelasma. Pre-school children were also investigated by Angelopoulou et al. [10]. In this case, the authors tested the efficacy of a simplified Pythagorean self-awareness intervention on heart rate variability parameters, perceived stress, and behaviors of children. They demonstrated the beneficial effect of such an intervention on the parameters tested, with a possible favorable repercussion on future unhealthy habits and non-communicable diseases. Furthermore, in another paper included in this Special Issue [11], the authors retrospectively analyzed the clinical impact of empirical therapy in children with vasovagal syncope. Specifically, Tao et al. showed how the efficacy of selected empiric therapies (e.g., orthostatic training, oral rehydration salts, metoprolol, or midodrine hydrochloride) in children was limited, further suggesting how individualized treatments need further dedicated studies. Changing the topic from persisting problems to new diagnostic techniques, a German research group published a study [12] on the association between two microRNA sequences (namely 126-3p and 126-5p) and aortic stiffness in patients affected by Turner syndrome. The authors described how, in a small cohort of Turner patients, these MiRNA were linked to significantly higher levels of aortic stiffness compared with age-matched volunteers.

Finally, a very hot topic was addressed in a paper from Taiwan [13], showing how viral infections (including COVID-19) may be an important trigger factor in the development of Kawasaki Disease and speculating on how non-pharmacological interventions (i.e., mask-wearing and social distancing) may play key roles in preventing not only viral infection in children but also the development of Kawasaki disease and its possible complications.

To summarize, the heterogeneity of the studies presented in this Special Issue, including advanced imaging or laboratory techniques (i.e., cardiac magnetic resonance, miRNA evaluation), old or new infective conditions (i.e., rheumatic heart disease, COVID-19), and the numerous types of congenital heart diseases, appropriately represents the diversity and magnitude of the research progress that pediatric cardiology has made and is still making. Importantly, the geographic origins of these 13 high-level papers suggest that the burden of treating pediatric cardiology diseases is a worldwide affair that needs an internationally coordinated effort to be properly addressed, starting with clinical research.
